# Screening and treatment of hypertension in older adults: less is more?

**DOI:** 10.1186/s40985-018-0101-z

**Published:** 2018-09-03

**Authors:** Daniela Anker, Brigitte Santos-Eggimann, Valérie Santschi, Cinzia Del Giovane, Christina Wolfson, Sven Streit, Nicolas Rodondi, Arnaud Chiolero

**Affiliations:** 1 Institute of Primary Health Care (BIHAM), University of Bern, Bern, Switzerland; 2 Institute of Social and Preventive Medicine (IUMSP), Lausanne University Hospital, Lausanne, Switzerland; 3 La Source, School of Nursing Sciences, University of Applied Sciences and Arts Western Switzerland, Lausanne, Switzerland; 4 Department of Epidemiology, Biostatistics and Occupational Health, McGill University, Montreal, Canada; 5 Department of General Internal Medicine, Inselspital, Bern University Hospital, University of Bern, Bern, Switzerland

**Keywords:** Screening, Hypertension, Older adults, Frailty

## Abstract

Screening and treatment of hypertension is a cornerstone of cardiovascular disease (CVD) prevention. Hypertension causes a large proportion of cases of stroke, coronary heart disease, heart failure, and associated disability and is highly prevalent especially among older adults. On the one hand, there is robust evidence that screening and treatment of hypertension prevents CVD and decreases mortality in the middle-aged population. On the other hand, among older adults, observational studies have shown either positive, negative, or no correlation between blood pressure (BP) and cardiovascular outcomes. Furthermore, there is a lack of high quality evidence for a favorable harm-benefit balance of antihypertensive treatment among older adults, especially among the oldest-old (i.e., above the age of 80 years), because very few trials have been conducted in this population. The optimal target BP may be higher among older treated hypertensive patients than among middle-aged. In addition, among frail or multimorbid older individuals, a relatively low BP may be associated with worse outcomes, and antihypertensive treatment may cause more harm than benefit. To guide hypertension screening and treatment recommendations among older patients, additional studies are needed to determine the most efficient screening strategies, to evaluate the effect of lowering BP on CVD risk and on mortality, to determine the optimal target BP, and to better understand the relationship between BP, frailty, multimorbidity, and health outcomes.

## Background

Screening and treatment of hypertension is a cornerstone of the prevention of cardiovascular disease (CVD), which is the leading cause of death and hospitalization worldwide [1]. Hypertension causes a large proportion of stroke, coronary heart disease, heart failure, and associated disability [2]. Currently, one quarter to one third of the adult population in the USA and in European countries have hypertension [3, 4]. Because blood pressure (BP) increases with age [5], and as a result of population aging [1], the prevalence of hypertension is expected to increase in the coming decades and a rapidly growing number of older adults will have to be managed for hypertension. This constitutes a true public health challenge for healthcare providers, who are in need of evidence-based clinical guidance.

While there is high quality evidence to guide screening and treatment of hypertension for the prevention of CVD in middle-aged adults, little evidence exists among older adults. Few trials have been conducted in this population [6–9], especially among the oldest-old, i.e., aged above 80 years. Further, there are conflicting observations about the relationship between BP and health outcomes among older adults, some cohort studies having shown that low BP could be detrimental especially among the oldest frail or multimorbid adults [10, 11].

In this review, we aimed to critically appraise evidence and guidelines for screening and treatment of hypertension in older patients, with an emphasis on the oldest-old, multimorbid, and frail patients. Due to the broadness of the field, we did not conduct a systematic review, better designed to address relatively narrow research questions, and rather preferred to conduct a narrative review, better suited to provide interpretation and critique of evidence [12]. First, we looked for data on how BP and hypertension relate to age, health outcomes, and mortality among older individuals. Second, we searched for experimental and observational evidence on the relationship between BP, antihypertensive treatments, and health outcomes in older adults, accounting for different health status, notably for frailty [13]. Finally, we critically appraised recommendations from major hypertension management guidelines for older adults.

## Epidemiology of blood pressure and hypertension-related mortality across ages

**Fig. 1:**
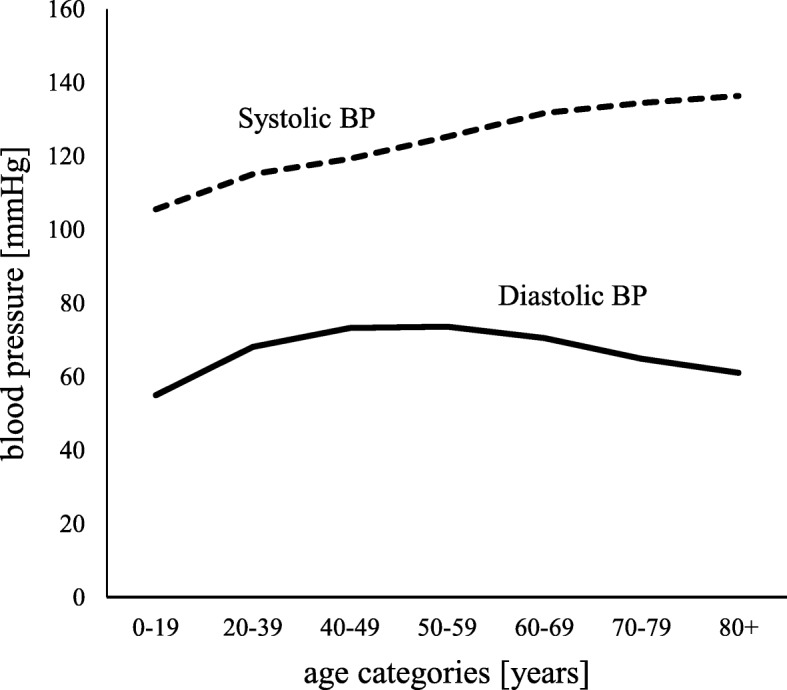
Mean SBP and DBP according to age. The data was extracted from the National Health and Nutrition Examination Survey held in the USA between 2013 and 2014. Source: Centers for Disease Control and Prevention [18, 68]

**Fig. 2:**
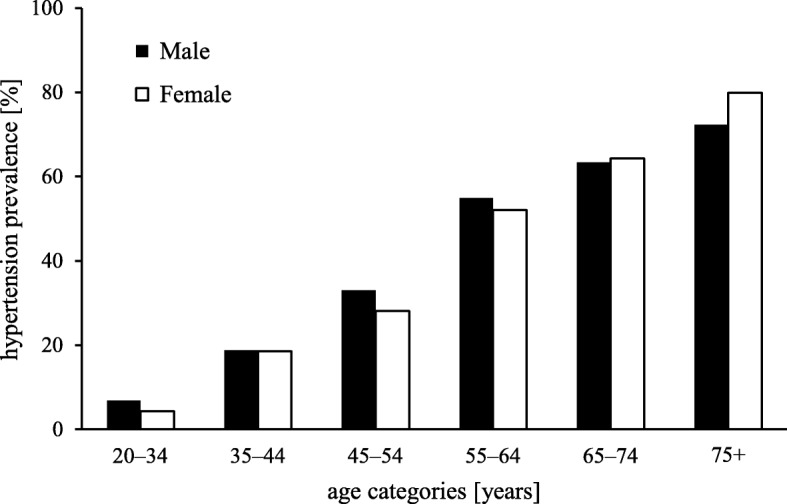
Hypertension prevalence according to sex in the USA over the years 2011 to 2014. The data was extracted from the National Health and Nutrition Examination Survey held in the USA between 2011 and 2014. Source: Centers for Disease and Prevention [14]

BP increases steadily with age [5], and consequently, the prevalence of hypertension is higher in older age groups [14]. Hypertension is diagnosed by sustained elevated BP, with a threshold usually set at 140/90 mmHg (based on office measurement) [6–9], although recent guidelines set the diagnostic threshold at lower BP levels [15]. In the USA and in European countries, one quarter to one third of the general population would have hypertension [3, 4]. Many epidemiologic studies have shown that systolic blood pressure (SBP) increases with age while diastolic blood pressure (DBP) increases up the age of 60–70 years and decreases with older age **(**Fig. 1**)** [16, 17]. In some countries, up to 75% of older adults are hypertensive [3, 14, 18] **(**Fig. 2**)**. Middle-aged adults most often develop systolo-diastolic hypertension, whereas older adults develop predominantly isolated systolic hypertension (defined as SBP ≥ 140 mmHg and a DBP < 90 mmHg) [19].

Hypertension is a major cause of CVD and mortality [20]. A large meta-analysis including 61 prospective studies and analyzing data from one million adults aged between 40 and 89 years demonstrated a strong positive log-linear relationship between BP and both cardiovascular and all-cause mortality across all age groups [5, 21]. Further, in all age groups, the risk was the lowest at 115/75 mmHg [5]. A log-linear relationship between BP and cardiovascular health outcomes was also observed in several other studies [21–23]. A key to better understand the relationship between BP and health outcomes among older adults is to account for the very high absolute risk of CVD in this population [21, 23]. This means that among older adults, a BP difference of 10 mmHg, for instance, is associated with a much larger difference in absolute risk of CVD compared to the same BP difference among younger adults [5]. Consequently, the potential absolute benefit of reducing BP could be much higher among older adults, and more so among oldest-old patients. The relationship between BP and cardiovascular health outcomes is however much more complex among the oldest-old, as discussed in detail below.

## Measurement of blood pressure and screening for hypertension in older adults

Methods of BP measurement in older adults are roughly similar to the methods in middle-aged adults, but closer monitoring is recommended by several guidelines [6, 8, 15], especially because older adults tend to have higher BP variability [24]. Older adults also have more frequently isolated systolic hypertension [19]. In the office, BP is measured traditionally on the upper arm using either the auscultatory method (requiring adequate training of the assessor) or the oscillometric method (requiring a clinically validated automated measurement device) [8]. To diagnose hypertension, repeated BP measurements at multiple visits are needed. Out-of-office measurements, either using ambulatory BP monitoring (ABPM) or home BP monitoring (HBPM) [7, 8], are recommended to confirm diagnoses of hypertension, especially in older adults. Orthostatic hypotension is frequent among older adults, with up to 30% among those aged more than 70 years old, and can seriously complicate treatment of hypertension [25, 26]. Recent guidelines recommend searching systematically for orthostatic hypotension in older individuals [8].

Screening for hypertension is justified for several reasons. In general, screening aims to distinguish apparently healthy persons who probably have a disease from those who probably do not [27]. In the case of hypertension, screening aims to identify individuals with sustained elevated BP and who would benefit from treatment through a reduction in CVD risk. According to the Wilson and Jungner criteria [27], screening for hypertension in older adults can be easily justified **(**Table 1**)** because hypertension is highly prevalent and causes serious health issues, BP measurement is relatively simple, accessible and acceptable for older adults, and treatments are available and efficient to lower BP.

**Table 1:** Selected Wilson and Jungner screening criteria adapted to hypertension screening in older adults [27]

Criterion	Criterion related to older adults
The condition sought should be an important health problem	Hypertension CVD and the absolute risk associated with elevated BP in older adults is very high. Hypertension is highly prevalent in the general population and the prevalence increases with age (reaching up to 75% among adults aged 75 years and more).
There should be an accepted treatment for patients with recognized disease	Treatments are accessible and have been shown to be effective among middle-aged adults. The evidence among older adults is much weaker. In frail or multimorbid patients, lowering BP could cause harm [10].
Facilities for diagnosis and treatment should be available	Screening and diagnosis are usually done by primary care physicians. Screening can also be done out of the office, e.g., by pharmacists [69]. Due to the growing number of older adults, provision of treatment will require growing resources, i.e., primary care physicians and other health professionals. To improve hypertension control, novel approaches to care are needed, e.g., team-based care [70].
There should be a recognizable latent or early symptomatic stage	Elevated BP is a causal risk factor for CVD. However, the discriminative power of BP measurement between high- and low-risk patients is weak [28]. Accordingly, other factors such as age and history of CVD are suggested to be more efficient for the assessment of the risk to develop CVD.
There should be a suitable test or examination	Auscultatory or oscillometric methods can be used. For the auscultatory method, training is necessary. For the oscillometric method, a clinically validated device should be used.
The test should be acceptable to the population	BP measurement is well accepted among older adults.
The natural history of the condition, including development from latent to declared disease, should be adequately understood	The relationship between BP and cardiovascular outcomes are not clearly defined in older adults, especially in frail or multimorbid older adults.

There are however two major issues with hypertension screening in older adults. A first issue is the weak discriminative power of elevated BP for having or not CVD later in life [28]. While individuals with elevated BP have a higher relative risk of CVD, many cases of CVD will occur in individuals with normal BP. This is especially problematic for patients with BP close to the threshold above which a treatment is recommended, and many older adults have BP close to such threshold. To overcome this issue, two approaches are possible. The first approach is a high-CVD risk prevention strategy, where the decision to treat hypertension is not based on BP level, but rather on an estimated absolute CVD risk [29]. The idea is that, for the same BP reduction, the absolute benefit will be much higher among patients at high absolute CVD risk compared with patients at relatively low absolute CVD risk. In a simulation study, Karmali et al. determined that a treatment strategy based on absolute CVD risk would reduce the proportion of treated individuals by 29%, while preventing 16% more events for the same number of persons treated, compared to a treatment strategy based on treating everyone with SBP higher than 150 mmHg [30]. The second approach would be a population-based prevention strategy shifting the distribution of BP toward lower levels in the entire population, instead of lowering BP only in high-risk populations with high BP or high risk of CVD [23, 31].

Apart from the weak discriminative power of elevated BP, a second issue with BP screening among older adults is that the benefit of lowering BP among the oldest-old is disputable. While these patients are at very high risk for CVD, there are doubts about a true benefit of BP lowering drugs on mortality and other health outcomes. Instead of being beneficial, a low BP might indeed be detrimental among oldest-old patients [10].

## Blood pressure and related outcomes: evidence from trials and cohort studies in oldest-old adults

The relationship between BP and mortality and other health outcomes in older age and the decision to treat or not oldest-old adults for hypertension is highly debated. In middle-aged adults, the risk for cardiovascular events, cardiovascular mortality, and all-cause mortality increases with increasing BP [5]. Likewise, numerous trials in middle-aged adults [32–35], and others like SHEP [36] and Syst-Eur [37], which enrolled participants 60 years and over, have shown that lowering BP with antihypertensive treatment reduces mortality and CVD risk.

In oldest-old adults, however, evidence about the predictive value of BP and the benefit-harm balance of treating hypertension is inconsistent [6–9]. On the one hand, two randomized controlled trials (RCTs)—SPRINT and HYVET—targeting older patients aged respectively 75 and 80 years old and over have shown the benefits of lowering BP by reducing mortality and CVD risk [38, 39]. On the other hand, several cohort studies of adults 80 years of age and over have shown that participants with low BP at baseline had higher all-cause mortality rates [40–50]. These findings suggest that lowering BP among the oldest-old might be harmful. Table 2 presents a detailed overview of these studies. Several cohort studies have also shown that low BP was associated with a decline in cognitive and physical abilities [51–54].

**Table 2:** Results from randomized controlled trials and cohort studies among oldest-old patients

Study acronym or first author, country, publication year	Population	Intervention or exposure BP category	Comparison or reference BP category	Outcomes (mortality and CVD)	Conclusion
Randomized controlled trials					
SPRINT, USA, 2016 [39]	≥ 75 years; *N* = 2636 Condition: hypertension but no diabetes	Intensive treatment: SBP targets < 120 mmHg	Standard treatment: SBP targets < 140 mmHg	HR (95% CI) for all-cause mortality: 0.67 (0.49–0.91) HR (95% CI) for composite CVD events (primary endpoint): 0.66 (0.51–0.85)	More intensive treatment among adults aged 75 years or older significantly reduced the rates of fatal and nonfatal major cardiovascular events and death from any cause, irrespective of frailty status
HYVET, Europe, China, Australasia, and Tunisia, 2008 [38]	80 years or older; *N* = 3845 Condition: sustained SBP of ≥ 160 mmHg	Active treatment	Placebo	HR (95% CI) for all-cause mortality: 0.79 (0.65–0.95) HR (95% CI) for fatal and nonfatal stroke (primary endpoint): 0.70 (0.49–1.01)	Active treatment in persons 80 years of age or older reduced the rate of death from any cause and cardiovascular events, irrespective of frailty status
Population-based observational studies					
Streit, the Netherlands, 2018 [50]	≥ 85 years; *N* = 570 Setting: population-based Leiden 85-plus cohort study	10 mmHg lower SBP	–	HR (95% CI) for all-cause mortality in participants with and without antihypertensive treatment: 1.29 (1.15–1.46) and 1.08 (1.00–1.18)	In persons aged 85 years and over, lower SBP was associated with higher all-cause mortality in participants prescribed antihypertensive therapy, irrespective of grip strength, used as a frailty indicator; in participants not prescribed antihypertensive therapy, there was no association between SBP and mortality
Ravindrarajah, UK, 2017 [42]	≥ 80 years; *N* = 144,403	SBP: (a) < 110, (b) 110–119, (c) 140–159, (d) ≥ 160 mmHg	SBP 120–139 mmHg (ref)	HR (95% CI) for all-cause mortality in treated fit women: (a) 1.86 (1.39–2.47), (b) 1.48 (1.23–1.79), (ref) 1, (c) 0.76 (0.70–0.84), and (d) 0.85 (0.75–0.96) HR (95% CI) for all-cause mortality in treated frail women: (a) 1.98 (1.53–2.56), (b) 1.44 (1.24–1.70), (ref) 1, (c) 0.80 (0.72–0.89), and (d) 0.97 (0.82–1.15)	In persons aged 80 years and over, lower SBP was associated with increased mortality rates, and lowest mortality rates were found in patients with baseline SBP between 140 and 159 mmHg; frail adults had higher mortality rates but the association with BP was similar compared with non-frail adults
Post Hospers, the Netherlands, 2015 [48]	≥ 80 years; *N* = 464 Setting: subpopulation of Longitudinal Aging Study Amsterdam	SBP (a) ≤ 120, b) > 140 mmHg; DBP (c) ≤ 70, (d) > 90 mmHg	SBP 121–140 mmHg (ref) DBP 71–90 mmHg (ref)	HR (95% CI) for all-cause mortality for SBP: (a) 1.16 (0.78–1.73), (ref) 1 and (b) 0.92 (0.71–1.20); HR (95% CI) for all-cause mortality for DBP: (c) 1.62 (1.23–2.14), (ref) 1, and (d) 0.94 (0.71–1.25)	In persons aged 80 years and over, low DBP was related to an increased all-cause mortality risk
Poortvliet, the Netherlands, 2013 [41]	≥ 90 years; *N* = 267 Setting: population-based Leiden 85-plus Study	SBP > 150 mmHg	SBP ≤ 150 mmHg	HR (95% CI) for all-cause mortality in participants with and without heart failure: 1.7 (1.2–2.3) and 2.0 (1.1–3.4)	In persons aged 90 years and over, low SBP was associated with increased mortality rates, irrespective of the presence or not of heart failure
Blom, the Netherlands, 2013 [44]	≥ 75 years; *N* = 851 Condition: without previous CVD; setting: subpopulation of prospective population-based Rotterdam study	SBP (a) 140–159/(b) ≥ 160 mmHg	SBP < 140 mmHg	HR (95% CI) for all-cause mortality in participants aged 75–84 years: (ref) 1, (a) 1.1 (0.9–1.3), and (b) 1.3 (1.0–1.6) HR (95% CI) for all-cause mortality in participants aged over 85 years: (ref) 1, (a) 0.7 (0.5–1.1), and (b) 0.7 (0.4–1.1)	After 75 years, high SBP is not associated with an increased mortality risk
Molander, Sweden, 2008 [40]	≥ 85 years; *N* = 5348	SBP (a) 121–140 /(b) 141–160/(c) > 160 mmHg	SBP ≤ 120 mmHg	HR (95% CI) for adjusted 4-year mortality: (ref) 1, (a) 0.44 (0.29–0.68), (b) 0.44 (0.29–0.68), and (c) 0.60 (0.37–0.96)	Low SBP was associated with increased mortality in persons aged 85 years and older; the optimal SBP for this age group could be > 140 mmHg
van Bemmel, the Netherlands, 2006 [43]	≥ 85 years; *N* = 571 Setting: population-based Leiden 85-plus Study	SBP (a) < 140 mmHg/(b) ≥ 160 mmHg	SBP 140–159 mmHg	RR (95% CI) for all-cause mortality: (a) 1.19 (0.79–1.79), (ref) 1, and (b) 0.66 (0.47–0.92)	BP < 140/70 mmHg was associated with excess mortality in persons aged 85 years and over
Satish, USA, 2001 [49]	≥ 85 years; *N* = 1088 Setting: subpopulation of cohort study	10 mmHg higher SBP and 10 mmHg higher DBP	SBP and DBP	HR (95% CI) of death with higher SBP in men: 0.92 (0.86–0.99) and in women: 1.00 (0.95–1.05) HR (95% CI) of death with higher DBP in men: 0.90 (0.80–1.02) and in women: 0.99 (0.89–1.10)	In men aged 85 years and older, higher SBP was associated with better survival
Guo, Sweden, 1997 [45]	≥ 75 years; *N* = 1810 Setting: community-dwelling	SBP (a) < 130/(b) ≥ 160 mmHg; DBP (c) < 75 (d) ≥ 95 mmHg	SBP ≥ 130 mmHg; SBP ≥ 75 mmHg	RR (95% CI) for death with SBP (a) 1.39 (1.11–1.73), (ref) 1, (b) 1.15 (0.97–1.37) and with DBP (c) 1.21 (1.02–1.43), (ref) 1, and (d) 0.91 (0.71–1.17)	In people aged 75 years and older, there was a marked increase in 5-year all-cause mortality with low BP (especially in participants with preexisting CVD, limitation in activities of daily living, and cognitive impairment)
Hakala, Finland, 1997 [46]	≥ 75 years; *N* = 521	10 mmHg higher SBP and 5 mmHg higher DBP	–	RR (95% CI) for higher SBP: 0.90 (0.85–0.96) RR (95% CI) for higher DBP: 0.92 (0.68–0.99)	Among subjects aged 75 years and over, high BP was associated with favorable 5-year survival
Mattila, Finland, 1988 [47]	≥ 85 years; *N* = 561 old people Setting: community-dwelling persons	SBP (a) < 120/(b) 120–139/(c) 140–159/(d) 160–179/(e) 180–199/(f) > 200 mmHg DBP (g) < 70/(h) 70–79/(i) 80–89/(j) 90–99/(k) 100–109/(l) > 110 mmHg	Mean survival rates in the Finnish population aged 85 years and over	5-year survival rates (SD) according to SBP level: (a) 0.22 (0.15), (b) 0.59 (0.16), (c) 1.08 (0.13), (d) 1.41 (0.14), (e) 1.32 (0.21), and (f) 1.49 (0.38) 5-year relative survival rates (SD) according to DBP level: (g) 0.72 (0.17), (h) 0.76 (0.18), (i) 1.13 (0.13), (j) 1.35 (0.14), (k) 1.19 (0.23), and (l) 1.54 (0.36)	The lowest survival was observed in individuals with the lowest SBP and DBP; survival was highest in subjects with BP ≥ 160/90 mmHg

*HYVET*, hypertension in the very elderly trial; *SPRINT*, systolic blood pressure intervention trial; *N*, number of participants; *BP*, blood pressure; *SBP*, systolic blood pressure; *DBP*, diastolic blood pressure; *HR*, hazard ratio, *CI*, confidence interval; ref., reference; *CVD*, cardiovascular disease

Several reasons could explain the discrepancies between the results from trials and from cohort studies, that is, (1) effect modification by frailty or other indicators of poor health, (2) confounding and reverse causality, and (3) selection of patients in trials **(**Table 3**)**.

**Table 3:** Hypotheses on discrepancies between cohort studies and RCTs

Hypotheses	Description
Effect modification of the relationship between BP and health outcomes by frailty or other indicators of poor health	The predictive effect of BP on mortality and adverse health outcomes might be reversed by age-related frailty or other indicators of poor health (e.g., multimorbidity) [11]. Several cohort studies, which enrolled participants aged 60 years and over, have found that participants with no indicators of poor health with high BP had higher mortality rates and worse health outcomes, while participants with indicators of poor health with high BP had lower mortality rates and better health outcomes. The association may be modified by frailty [58, 59], limitations in cognitive and physical functioning [45, 48], and multimorbidity [49].
Confounding and reverse causality	Confounding: the relationship can be confounded by unmeasured factors, which have an effect on both BP and the risk of adverse health outcomes. Reverse causality: some conditions, which can be initially caused by high BP, evolve to become the cause of low BP [42].
Patient selection in clinical trials	RCTs might select participants in better health, with fewer comorbidities, and with a longer life expectancy than participants in population-based cohort studies, with the latter being more representative of the general population. For instance, HYVET and SPRINT trials have well-defined and restrictive eligibility criteria for participants, who are healthier than the general population of the same age [10]. Post-randomization confounding may also bias results of trials [62].

First, the association between BP and CVD risk may be modified by poor health, such as frailty [11]. Frailty is a multidimensional geriatric syndrome characterized by increased vulnerability and loss of adaptability to stress [55–57] **(**Table 4**)**. For instance, in a study by Van Hateren et al., frail participants with high BP had lower all-cause mortality rates than frail participants with low BP [58]. In another study by Odden et al. walking speed—used as a frailty indicator—modified the association between BP and the risk of CVD [59]. Among slow walkers, high BP was not associated with higher cardiovascular mortality rates, while high BP was associated with higher CVD mortality rates among fast walkers. Other studies have shown that the relationship between BP and both mortality and adverse health outcomes could be modified by poor health, such as physical and mental impairment [45, 48]. In the two previously mentioned RCTs, HYVET and SPRINT, post hoc analyses however suggested that frailty does not modify the benefit of anti-hypertensive treatment [39, 60].

**Table 4:** Definition of frailty

*General definition* Frailty is a multidimensional geriatric syndrome characterized by an increased vulnerability and a loss of adaptability to stress. This state is characterized by an increased risk of adverse outcomes, such as falls, delirium, disability, and mortality [55, 57, 71]. Two main models allow to assess the frailty status of patients: Fried’s phenotype model and Rockwood’s cumulative deficit model.	
*Fried’s phenotype model* A frailty phenotype is based on the five following features: • Unintentional weight loss • Self-reported exhaustion • Low energy expenditure • Slow gait speed • Weak grip strength Patients with none of these features are considered as not frail (or robust), those with one or two as pre-frail, and those with three or more as frail [13].	
*Rockwood’s cumulative deficit model* Frailty is defined as an accumulation of defined individual deficits, where the more of these deficits a person has, the higher the probability that this person is frail. Accordingly, a “frailty index” can be calculated from the addition of relevant age-related health variables such as symptoms, signs, abnormal laboratory values, disease states, and disabilities [72].	

The modification of the association between BP and health outcomes in case of frailty or poor health may be explained by physiological reasons. Low BP in old and frail individuals might indeed cause hypo-perfusion of vital organs and accelerate physical and mental decline [11], a state of physiological weakness, which is often worsened by the presence of polypharmacy and comorbidity [10, 53]. Some authors suggest that the presence of frailty or other indicators of poor health might be an argument for deprescribing antihypertensive treatments (Table 5) [10, 24].

**Table 5:** Deprescribing antihypertensive treatment

*Definition of deprescribing* Deprescribing can be defined as a systematic process of gradually lessening or stopping drugs with the aim to reduce polypharmacy and improve patient outcomes. This process implies the identification of drugs that are suspected to induce no benefit, or which potentially cause more harm than benefit for the patient. Polypharmacy is common in older adults, and they are especially vulnerable to drug-related adverse events [73]. Deprescribing is therefore particularly relevant in older adults.	
*Deprescribing antihypertensive treatment in older adults* Some data on antihypertensive treatment withdrawal exist showing that among well-selected older adults [74], a relevant proportion of patients stay normotensive [75, 76], with minor withdrawal-associated risks [77] and potentially beneficial effects on health [78]. Accordingly, a recent Canadian Guideline, specifically addressing BP management in frail older adults, recommends to generally prescribe no more than two antihypertensive medications and to reduce antihypertensive treatment when systolic BP is below 140 mmHg [79]. A recent Cochrane review concluded that the effect of deprescribing was uncertain, with however no increase in mortality among participants allocated to withdrawing from antihypertensive therapy [80].	

**Fig. 3:**
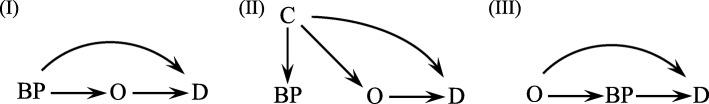
The hypothetical causal relationship between blood pressure (BP) and related adverse health outcomes or mortality according to different scenarios is depicted in these graphs. (I) Usually, BP has a causal effect on adverse health outcomes (O) and death (D). (II) The relationship can be confounded by unmeasured factors (C), which have an effect on both BP and the risk of adverse health outcomes. (III) In a situation of reverse causality, some conditions, which can be initially caused by high BP, evolve to become the cause of low BP

Second, the association of a lower BP with worse health outcomes in cohort studies may be due to confounding and reverse causality (Fig. 3). On the one hand, the association between BP and any adverse health outcomes may be confounded by some unmeasured factors. On the other hand, in a case of reverse causality, some conditions, which can be initially caused by high BP, evolve to become the cause of low BP. For example, high BP is a cause of heart failure [61], but in an advanced stage, heart failure becomes the cause of a lower BP. In this case, there is an association between low BP and the condition, as well as between low BP and mortality, but both these associations are the result of a reverse causality. This situation is frequent in circumstances of end-of-life care [42]. Finally, the selection of relatively healthy patients in randomized trials compared with cohorts may be a third explanation of the discrepancies between RCTs and cohort studies [10]. Post-randomization confounding may also bias results of RCTs [62].

To disentangle these complex controversies on the association between BP and health outcomes among older adults, hypertension trials targeting specifically frail or multimorbid older adults are needed.

## International guidelines for hypertension management in older adults

International guidelines about screening for and treatment of hypertension are mainly targeting middle-aged adults (40–60 years). In line with the lack of strong evidence on the most adequate way of managing hypertension in older adults, especially over 80 years of age, there are no firm and consistent recommendations for this population (Table 6).

**Table 6:** Recommendations for older adults from recent international guidelines on screening for and treatment of hypertension

	NICE, 2011	ESH/ESC, 2013	JNC 8, 2014	USPSTF, 2015	ACC/AHA, 2017
Measurement and screening	If normal BP: screening every 5 years; if BP close to 140/90 mmHg: screening more frequently ABPM or HBPM for monitoring treatment effect No specific recommendations for older adults	No mention of screening Out-of-office BP is recommended to search for orthostatic hypotension, especially for older adults, and for white coat and masked hypertension	No recommendations about measurement or screening	If 18–39 years with normal BP (< 130/85 mmHg) without other risk factors: screening every 3 to 5 years; if ≥ 40 years or at increased risk for high BP: annual screening No specific recommendations for older adults	No mention of screening Out-of-office BP is recommended to adapt BP-lowering medication and to screen for white coat and masked hypertension
Target BP and/or treatment	Under 80 years: 140/90 mmHg; 80 years and more: 150/90 mmHg 80 years and more: same drug regimen than for people aged 55–80 years, accounting for comorbidities	In older adults < 80 years with SBP ≥ 160 mmHg: 140–150 mmHg • If fit: SBP < 140 mmHg may be considered • If fragile: adapting to individual tolerability In older adults > 80 years with SBP ≥ 160 mmHg: • If in good physical and mental conditions: SBP 140–150 mmHg (may be set high if no history of CVD) • Frail: leave decisions to the treating physician, based on monitoring of the clinical effects of treatment	30–59 years: 140/90 mmHg; 60 years and more: 150/90 mmHg (some experts recommend 140 mmHg). No need to adapt treatment if SBP is lower than 140 mmHg and if there are no adverse effects on health or on quality of life No specific recommendations for older adults	Younger adults: 140/90 mmHg; 60 years and more: 150/90 mmHg (according to some expert opinion: 140/90 mmHg) No specific recommendations for older adults	Same treatment targets than in younger adults (130/80 mmHg) with close monitoring of BP and treatment effect in case of comorbidity NB. For the primary prevention of CVD, treatment is recommended in adults with an estimated 10-year ASCVD risk of ≥ 10% and SBP ≥ 130/80 mmHg. Hence, because the majority of older adults have a 10-year ASCVD risk ≥ 10% and have BP ≥ 130/80 mmHg, a treatment is recommended

*ASCVD*, atherosclerotic cardiovascular disease; *BP*, blood pressure; *SBP*, systolic blood pressure; *DBP*, diastolic blood pressure; *CVD*, cardiovascular disease; *NICE 2011*, UK National Institute for Health and Care Excellence Guidelines 2011 [6]; *JNC 8 2014*, the Eighth Joint National Committee Guidelines 2014 [7]; *ESH/ESC 2013*, European Society of Hypertension and the European Society of Cardiology Guidelines 2013 [8]; *ACC/AHA 2017*, American College of Cardiology and American Heart Association Guideline 2017 [15]; *USPSTF 2015*, US Preventive Services Task Force Guidelines 2015 [9]

The US Preventive Services Task Force (USPSTF) 2015 guidelines recommend screening for high BP among all adults aged 18 years or more [9]. The USPSTF does however not give any specific recommendation for older adults regarding screening and BP measurement. The European Society of Hypertension and the European Society of Cardiology (ESH/ESC) 2013 guidelines recommend out-of-office BP measurement in older adults to search for orthostatic hypotension [8]. Other international guidelines do not give any specific recommendations related to screening for hypertension among older adults [6–8] (Table 6).

Most guidelines recommend higher BP targets among older adults compared with middle-aged adults [6–9]. Further, some of these guidelines recommend taking into account health status to set the targeted BP [6, 8]. The USPSTF 2015 guidelines and the Eighth Joint National Committee (JNC 8) 2014 guidelines recommend targeting 150/90 mmHg from age 60 years, but they mention that, according to some expert opinion, 140/90 mmHg can be targeted, as in younger adults [7, 9]. The UK National Institute for Health and Care Excellence (NICE) 2011 guidelines recommend targeting 150/90 mmHg from age 80 years, although 140/90 mmHg can be targeted among patients without comorbidities [6]. The ESH/ESC guidelines 2013 recommend targeting an SBP < 140 mmHg in fit older adults up to 80 years of age and tailoring BP target according to individual tolerability in frail patients. Above 80 years of age, for patients in good physical and mental condition, the ESH/ESC guidelines 2013 recommend targeting an SBP of 140–150 mmHg. For frail patients, the decision whether or not to treat has to be evaluated by the physician, with a close monitoring of clinical effects of treatment [8].

The recent American College of Cardiology (ACC)/American Heart Association (AHA) guidelines 2017 recommend a more aggressive treatment compared with other guidelines [15]. The BP threshold to define hypertension was lowered to 130/80 mmHg, including for patients over 80 years of age [15]. However, a close BP monitoring in older persons with high comorbidity is recommended. This major change in the definition of hypertension is mainly based on the results from the SPRINT trial [39]. These new ACC/AHA guidelines received critiques from experts, questioning SPRINT’s methodology [63] and its generalizability [64]. Some experts consider that these guidelines downplay the adverse effects of antihypertensive treatment, such as falls due to hypotension [65] and the risk for overtreatment and polypharmacy [10, 66], especially in frail oldest-old patients [65]. In reaction, some experts have developed their own guidelines with more conservative treatment recommendations accounting for detrimental effects of low BP reported in cohort studies [67].

In summary, while international guidelines give clear recommendations about screening for and treatment of hypertension in middle-aged adults, the recommendations in older adults are much less clear, with large inconsistencies across different guidelines. This reflects the lack of clear evidence on the risks and benefits of BP treatment and on the optimal BP targets in older adults, especially for the frail and oldest-old patients.

## Conclusion

Due to population aging, hypertension in older adults is a major and growing burden for the health care system. Screening and treatment of hypertension in older adults, and especially in the oldest-old, is still a matter of intense debate notably due to the lack of high quality evidence. Several important research questions have still to be addressed **(**Table 7**)**, especially about the relationship between frailty, multimorbidity, BP, and associated health outcomes. While screening and treatment strategies accounting for general health status or frailty are appealing, it raises the question of how to assess these clinical features with confidence [56]. More broadly, various screening and treatment strategies should be evaluated and compared with a population-based approach aiming to find the most appropriate way of managing hypertension in older adults.

**Table 7:** Challenges and areas for research about screening and treatment of hypertension among older adults

1. How to screen for hypertension among older adults? At what frequency? In which setting? Universal vs targeted screening?	
2. What are the benefits and harms of lowering BP among older adults? What is the effect notably on the quality of life?	
3. What are the characteristics of the oldest-old whose high BP is associated with favorable health outcomes?	
4. What is the relationship between frailty and BP, and what are the consequences of this relationship on the prescription of treatment?	
5. Can some factors (frailty, multimorbidity, polypharmacy, orthostatic hypotension) in older age be an argument for deprescribing of antihypertensive drugs?	

## Abbreviations

ABPM: ambulatory BP monitoring
ACC/AHA 2017: American College of Cardiology and American Heart Association
ASCVD: Atherosclerotic cardiovascular disease
BP: Blood pressure
CVD: Cardiovascular diseases
DBP: Diastolic blood pressure
ESH/ESC: the European Society of Hypertension and the European Society of Cardiology
HBPM: Home BP monitoring
JNC 8: Eighth Joint National Committee
NICE: National Institute for Health and Care Excellence
SBP: Systolic blood pressure
USPSTF: US Preventive Services Task Force
